# *Ageratum conyzoides* Extract Promotes Hair Shaft Thickness Accompanied by Increased Growth Factor Levels and Enhanced Antioxidant Status in C57BL/6 Mice

**DOI:** 10.3390/cimb48101076

**Published:** 2026-10-18

**Authors:** Chunsik Yi, Jong-Hwan Lim

**Affiliations:** InnoTNB Co., Ltd., 815, 8, Beobwon-ro 8-gil, Songpa-gu, Seoul 05855, Republic of Korea; ycs321@innotnb.co.kr

**Keywords:** *Ageratum conyzoides*, hair follicle, hair regrowth, hair shaft thickness, cuticle integrity, oxidative stress, antioxidant status, VEGF, IGF-1

## Abstract

Hair loss results from disrupted hair follicle cycling, age-related hormonal imbalance, and oxidative stress, and remains a significant psychological burden for which safe, evidence-based interventions are limited. This study evaluated the effects of a standardized *Ageratum conyzoides* extract (ACE) on hair follicle growth, hair shaft thickness, and hair shaft morphology in C57BL/6 mice. Mice received oral ACE (10, 20, or 50 mg/kg) for 21 days, and hair regrowth, follicle and shaft characteristics, antioxidant-related responses, serum IGF-1 and VEGF levels, and cuticle morphology were evaluated. ACE accelerated hair regrowth at 20 and 50 mg/kg, increased hair shaft thickness and follicle diameter across all tested doses, and increased follicle number at 20 and 50 mg/kg compared with vehicle, with effects most consistent at 50 mg/kg. ACE upregulated Nrf2, HO-1, SOD2, and GPx1 expression in dorsal skin, increased serum IGF-1 and VEGF levels and dorsal skin superoxide dismutase (SOD) activity, and reduced dorsal skin malondialdehyde (MDA). Scanning electron microscopy revealed reduced hair shaft cuticle damage scores in ACE-treated mice, indicating improved cuticle surface integrity. These findings indicate that ACE promotes hair follicle growth and increases hair shaft thickness, accompanied by increased growth factor levels and enhanced antioxidant status, thereby extending prior findings and supporting further investigation of ACE for hair health.

## 1. Introduction

Hair loss is a multifactorial condition associated with disrupted hair follicle (HF) cycling, hormonal imbalance, and oxidative stress, and can impose a substantial psychological burden [1,2,3]. HF growth is regulated through interactions between dermal papilla and epithelial cells during the anagen, catagen, and telogen phases, and premature transition from anagen to telogen contributes to hair loss [2,4,5]. Oxidative stress may further impair follicular function, while growth factors such as vascular endothelial growth factor (VEGF) and insulin-like growth factor-1 (IGF-1) are important for follicular proliferation and vascular support [3,6].

Current pharmacological options, including finasteride and minoxidil, are effective yet limited by adverse side effects during long-term use [7,8]. Thus, natural alternatives rich in antioxidants that foster both hair growth and overall hair health have gained significant attention [9,10]. *Ageratum conyzoides* L. contains diverse bioactive constituents, including alkaloids, flavonoids, chromenes, benzofurnas, and terpenes [11,12], and has recently been shown to exert hair-growth-promoting effects [13]. In our previous study, a standardized *Ageratum conyzoides* extract (ACE) enhanced antioxidant and hair-growth-related factors in human follicle dermal papilla cells (HFDPCs). Oral ACE administration in C57BL/6 mice also increased hair follicle number and depth and keratin expression compared with vehicle treatment, with topical minoxidil used as a positive control [14]. However, whether these cellular observations are reflected in vivo and whether ACE improves hair shaft structure have not been fully investigated.

While previous studies primarily focused on the cellular mechanisms and hair-growth effects of ACE, the present study investigated hair regrowth, hair shaft thickness, hair follicle number and diameter, antioxidant-related gene expression, SOD activity and MDA levels in dorsal skin, circulating IGF-1 and VEGF levels, and hair shaft morphology and cuticle integrity using scanning electron microscopy (SEM) in C57BL/6 mice. By integrating these biochemical and structural assessments, we aimed to determine whether the previously reported effects of ACE were accompanied by corresponding in vivo biochemical responses and structural changes in newly formed hair shafts.

## 2. Materials and Methods

### 2.1. Standardized Ageratum conyzoides Extract

Standardized *Ageratum conyzoides* extract was supplied by Gencor Pacific (Hong Kong, China; Lot No. AGE/23002). The extract was standardized using 5′-methoxynobiletin as a marker compound, with a specification of 0.33–0.50%, using a validated high-performance liquid chromatography-photodiode array (HPLC-PDA) method [15]. Briefly, chromatographic separation was performed on a SunFire C_18_ column (250 × 4.6 mm, 5 μm; Waters Corporation, Milford, MA, USA) maintained at 35 °C, using 0.1% formic acid in distilled water (A) and acetonitrile (B) as mobile phases under gradient elution. The flow rate was 0.8 mL/min, the injection volume was 10 μL, and detection was performed at 330 nm. Detailed chromatographic conditions are summarized in Supplementary Figure S1 and Table S1.

### 2.2. Animals

Six-week-old male C57BL/6 mice weighing 18–20 g were obtained from Orientbio (Seongnam, Republic of Korea). Before the experiment, the animals were acclimated for 7 days under controlled environmental conditions of 22 ± 3 °C, 50 ± 5% humidity, and a 12-h light/dark cycle. All experimental procedures were approved by the Institutional Animal Care and Use Committee (IACUC) of Chungnam National University (Approval No. 202403A-CNU-038).

### 2.3. Hair Regrowth Assessment

To ensure synchronization of the hair follicle cycle, mice exhibiting a homogeneous telogen-phase skin color were initially selected following acclimatization. Two weeks later, the dorsal hair was shaved, and mice showing a uniform dorsal skin appearance were randomly allocated to the experimental groups. The mice were administered daily via oral gavage for 21 days with distilled water as the vehicle control or ACE at 10, 20, and 50 mg/kg (*n* = 6 per group). No formal a priori sample-size calculation was performed. The sample size was chosen with reference to previous studies in the literature using similar mouse hair-growth models. Macroscopic changes in dorsal skin were photographed on days 1, 8, 15, and 22. Hair regrowth was evaluated based on skin color tone shifting from pink (score 0; no growth) to gray (score 1; early stage), dark gray (score 2; mid stage), and black (score 3; full coverage). The overall hair regrowth score was calculated by integrating the relative area ratio of each color score [16]. Hair thickness was measured in five hair shafts per mouse using microscopic images captured at 400× magnification and analyzed with ImageJ version 1.54t (National Institutes of Health, Bethesda, MD, USA). The average value per mouse was used for statistical analysis.

### 2.4. Histological Analysis

Dorsal skin samples were fixed in 4% neutral buffered formalin, processed for paraffin embedding, cut into 4-μm sections, and stained with hematoxylin and eosin (H&E). Hair follicle number and diameter were determined from at least three randomly selected fields per mouse at 100× magnification with ImageJ version 1.54t (National Institutes of Health, Bethesda, MD, USA).

### 2.5. Scanning Electron Microscopy (SEM) and Hair Shaft/Cuticle Damage Analysis

Three hair samples randomly collected from each mouse were analyzed using scanning electron microscopy (SEM3200, CIQTEK, Hefei, China) to characterize cuticle surface morphology. Hair surface cuticle score was evaluated from SEM images using a previously described five-point grading scale [17]: Grade 1, intact/undamaged hair; Grade 2, irregularly overlaid, visible, and lifted cuticles; Grade 3, severely lifted and peeled cuticles; Grade 4, partially exposed cortex; and Grade 5, exposed cortex without a cuticle layer, where higher scores indicated greater damage. Grading was performed by an investigator blinded to treatment group allocation.

### 2.6. Quantitative Real-Time PCR (qPCR)

Dorsal skin samples from the mice were homogenized with TRIzol reagent (Invitrogen, Thermo Fisher Scientific, Waltham, MA, USA), and total RNA was isolated following the manufacturer’s protocol. RNA concentration and purity were evaluated spectrophotometrically. Equal quantities of total RNA were then reverse-transcribed to cDNA (RevertAid Kit, Thermo Fisher Scientific, Waltham, Ma, USA). SYBR Green-based qPCR was used to quantify the mRNA levels of the antioxidant-related genes nuclear factor erythroid 2-related factor 2 (Nrf2), heme oxygenase-1 (HO-1), superoxide dismutase 2 (SOD2), and glutathione peroxidase 1 (GPx1). β-Actin served as the internal reference for normalization. Relative mRNA expression was determined by the 2^−ΔΔCt^ method and reported as fold change versus the vehicle control group.

### 2.7. Quantification of IGF-1, VEGF, and Oxidative Stress Markers

At the end of the experiment, blood was collected and centrifuged to obtain serum, which was kept at −80 °C until analysis. Serum IGF-1 and VEGF concentrations were measured with commercially available mouse-specific ELISA kits (Abcam, Cambridge, UK) following the manufacturers’ instructions. Dorsal skin samples were homogenized in ice-cold RIPA buffer (Thermo Fisher Scientific, Waltham, MA, USA) and centrifuged, after which the supernatants were used for biochemical analyses. Protein concentration in each supernatant was measured by the Bradford assay (Bio-Rad, Hercules, CA, USA) for normalization. Tissue MDA was quantified as a measure of lipid peroxidation using a thiobarbituric acid reactive substances (TBARS) Assay Kit (Cayman Chemical, Ann Arbor, MI, USA), whereas tissue SOD activity was determined using a Superoxide Dismutase Assay Kit (Cayman Chemical, Ann Arbor, MI, USA).

### 2.8. Statistical Analysis

Group differences were analyzed by one-way analysis of variance (ANOVA), followed by Dunnett’s multiple-comparison test. Statistical significance was defined as *p* < 0.05. Analyses were conducted using Origin 7 software (OriginLab Corporation, Northampton, MA, USA).

## 3. Results

### 3.1. ACE Promotes Hair Regrowth in C57BL/6 Mice

To evaluate the hair growth-promoting effect of ACE, dorsal skin color changes were monitored macroscopically following oral administration of vehicle or ACE (Figure 1a). Progressive skin darkening, indicative of anagen-associated pigmentation and hair regrowth, was observed in all groups over the study period. However, ACE-treated mice showed an earlier and more pronounced transition from pink to gray and dark gray coloration than vehicle-treated mice, particularly at 20 and 50 mg/kg. By day 15, pigmentation and hair regrowth were more advanced in the ACE-treated groups, with the greatest changes observed at 50 mg/kg. By day 22, the ACE-treated groups showed near-complete dorsal hair coverage, whereas hair regrowth remained less complete and more heterogeneous in the vehicle group. Consistent with these macroscopic observations, the hair regrowth score on day 15 was significantly higher in the ACE 20 and 50 mg/kg groups than in the vehicle group (*p* < 0.05; Figure 1b).

Hair thickness was significantly increased in all ACE-treated groups relative to vehicle (*p* < 0.05; Figure 1c). Similarly, histological analysis of dorsal skin sections showed that hair follicle number was significantly increased in the ACE 20 mg/kg and ACE 50 mg/kg groups compared with vehicle (*p* < 0.05; Figure 1d). Hair follicle diameter was significantly increased across all ACE-treated groups relative to vehicle (*p* < 0.05; Figure 1e). Together, these findings indicate that oral ACE administration accelerates hair regrowth and promotes hair follicle growth and hair shaft thickening in C57BL/6 mice.

### 3.2. ACE Upregulates Antioxidant-Related Gene Expression in Dorsal Skin

To investigate whether the hair growth-promoting effects of ACE were accompanied by changes in antioxidant-related gene expression, mRNA expression of Nrf2, HO-1, SOD2, and GPx1 was measured in dorsal skin tissue by qPCR (Figure 2). Nrf2 mRNA expression was significantly increased only in the ACE 50 mg/kg group compared with vehicle (*p* < 0.05; Figure 2a). A similar pattern was observed for HO-1, a downstream target of Nrf2, with a significant increase restricted to the ACE 50 mg/kg group (*p* < 0.05; Figure 2b). SOD2 mRNA expression was significantly elevated in both the ACE 20 mg/kg and ACE 50 mg/kg groups relative to vehicle (*p* < 0.05; Figure 2c), and GPx1 mRNA expression showed a comparable dose-dependent pattern, with significant increases at ACE 20 and ACE 50 mg/kg (*p* < 0.05; Figure 2d). These results indicate that ACE upregulates antioxidant-related gene expression in dorsal skin, with significant increases in SOD2 and GPx1 at 20 and 50 mg/kg and in Nrf2 and HO-1 at 50 mg/kg.

### 3.3. ACE Improves Hair Shaft Cuticle Integrity

To assess whether ACE affects hair shaft surface structure, cuticle morphology was examined by SEM and graded using a 5-point damage scale (Figure 3). Representative SEM images showed progressively smoother, more regularly overlapping cuticle scales with increasing ACE dose, whereas the vehicle group displayed more irregular, lifted, and disorganized cuticle edges (Figure 3a). Consistent with this qualitative observation, the hair surface cuticle score was significantly reduced in the ACE 50 mg/kg group compared with vehicle (*p* < 0.05), indicating improved cuticle integrity (Figure 3b). These findings indicate improved hair shaft cuticle integrity at the highest tested dose.

### 3.4. ACE Increases Serum Growth Factor Levels and Enhances Antioxidant Status

To determine whether the hair growth-promoting effects of ACE are associated with changes in circulating growth factor levels and local antioxidant status, serum IGF-1 and VEGF levels and dorsal skin MDA levels and SOD activity were measured (Figure 4). Serum IGF-1 levels were significantly increased in the ACE 50 mg/kg group compared with vehicle (*p* < 0.05; Figure 4a). Serum VEGF levels showed a dose-dependent increase, reaching statistical significance only in the ACE 50 mg/kg group (*p* < 0.05; Figure 4b). Dorsal skin MDA, a marker of lipid peroxidation, was significantly decreased in the ACE 50 mg/kg group relative to vehicle (*p* < 0.05; Figure 4c). SOD activity was also significantly increased in the ACE 50 mg/kg group compared with vehicle (*p* < 0.05; Figure 4d). Collectively, these results indicate that ACE increases circulating growth factor levels while enhancing antioxidant capacity and reducing lipid peroxidation in dorsal skin, predominantly at the highest dose tested.

## 4. Discussion

Hair loss is a multifactorial process involving dysregulation of hair follicle cycling, hormonal signaling, and oxidative stress [2,3,4,5]. In our previous cellular and preliminary animal studies, standardized ACE was shown to modulate 5α-reductase, estrogen receptor signaling, Wnt/β-catenin, and mitogen-activated protein kinase (MAPK) pathways, while increasing antioxidant enzymes in HFDPCs [14]. However, the previous study did not evaluate in vivo antioxidant responses in dorsal skin, circulating growth factor levels, or hair shaft microstructure and cuticle integrity. The present study therefore extends these findings by evaluating antioxidant-related responses in dorsal skin, circulating IGF-1 and VEGF levels, and hair shaft morphology and cuticle integrity in ACE-treated mice.

Oxidative stress is implicated in impaired hair follicle function through oxidative damage and premature senescence of dermal papilla cells, which may adversely affect follicular cycling and hair production [3,18]. Consistent with the increased SOD1, SOD2, and GPx protein expression previously observed in ACE-treated HFDPCs [14], oral ACE administration increased SOD2 and GPx1 mRNA expression in dorsal skin, reaching statistical significance at 20 and 50 mg/kg. Nrf2 and HO-1 expression levels were significantly increased at 50 mg/kg. In parallel, ACE treatment increased SOD activity and reduced MDA levels in dorsal skin at the highest dose. Together, these findings provide in vivo evidence of an enhanced antioxidant-related response and reduced lipid peroxidation in ACE-treated skin, thereby extending the antioxidant activity previously demonstrated in cultured follicular cells [14].

ACE administration also increased serum IGF-1 and VEGF, both of which were significantly elevated at 50 mg/kg. This is broadly consistent with our earlier in vitro observation that ACE increased VEGF and IGF-1 transcripts in HFDPCs, potentially involving Wnt/β-catenin pathway activation and IGF-1–GSK-3β–β-catenin crosstalk [14]. IGF-1 and VEGF have established roles in supporting follicular growth and vascularization, respectively [6,19,20]. In the present study, their increases coincided with improvements in hair regrowth, follicle number and diameter, and hair shaft thickness. These parallel responses suggest a possible association between ACE-induced growth factor responses and follicular growth. However, further studies involving pathway-specific inhibition or gene modulation are needed to clarify the underlying mechanisms.

Hair shafts from ACE-treated animals were thicker, and examination of their surface morphology showed more regularly arranged cuticular scales and fewer lifted or disorganized cuticle margins. Importantly, the present study extends our previous findings by demonstrating structural changes in newly formed hair shafts. Consistent with these observations, the hair surface cuticle score was significantly reduced at 50 mg/kg. Because the cuticle forms the outer protective layer of the hair fiber [21], preservation of its organization provides structural evidence that the effects of ACE are not restricted to follicular growth but may also extend to the morphology of the resulting hair shaft. This finding complements the increased keratin-1 and keratin-2 expression previously observed in ACE-treated mouse skin [14], although a direct mechanistic relationship between keratin expression and the improved cuticle morphology cannot be established from the present data.

The hair shaft findings should nevertheless be interpreted within the limitations of the experimental approach. The SEM-based damage score is a semi-quantitative morphological assessment and does not directly measure functional properties of the hair fiber [21,22]. In particular, hair gloss depends on optical reflectance from the fiber surface, whereas elasticity and mechanical strength require tensile measurements [21]. Accordingly, the increased shaft thickness and improved cuticle organization observed here should be considered evidence of improved hair shaft structural integrity rather than direct evidence of enhanced hair gloss or elasticity. Instrumental gloss measurements and tensile testing will be required to determine whether these morphological changes translate into functional improvements in hair properties.

Although the C57BL/6 mouse model is widely used to investigate hair follicle biology and hair growth, differences in hair follicle structure, hair cycling, and physiological regulation between mice and humans limit the direct translation of findings from this model to human hair growth outcomes [23,24]. In addition, the absence of a positive control, such as topical minoxidil, precludes direct comparison of the hair growth-promoting effects of ACE with those of established treatments. Furthermore, the morphological improvements observed in mouse hair shafts may not necessarily translate into improvements in human hair quality. Therefore, further clinical studies are warranted to determine whether oral ACE supplementation improves human hair health, particularly hair gloss, elasticity, and other relevant hair quality parameters.

## 5. Conclusions

Oral ACE promoted hair follicle growth and increased hair shaft thickness in C57BL/6 mice, accompanied by increased IGF-1 and VEGF levels, enhanced antioxidant status, and reduced lipid peroxidation. These findings extend our previous cellular observations by providing complementary in vivo evidence and demonstrate structural improvements in hair shaft thickness and cuticle integrity. Further studies are warranted to clarify the underlying mechanisms and determine whether these structural changes translate into improved hair gloss and elasticity in humans.

## Figures and Tables

**Figure 1:**
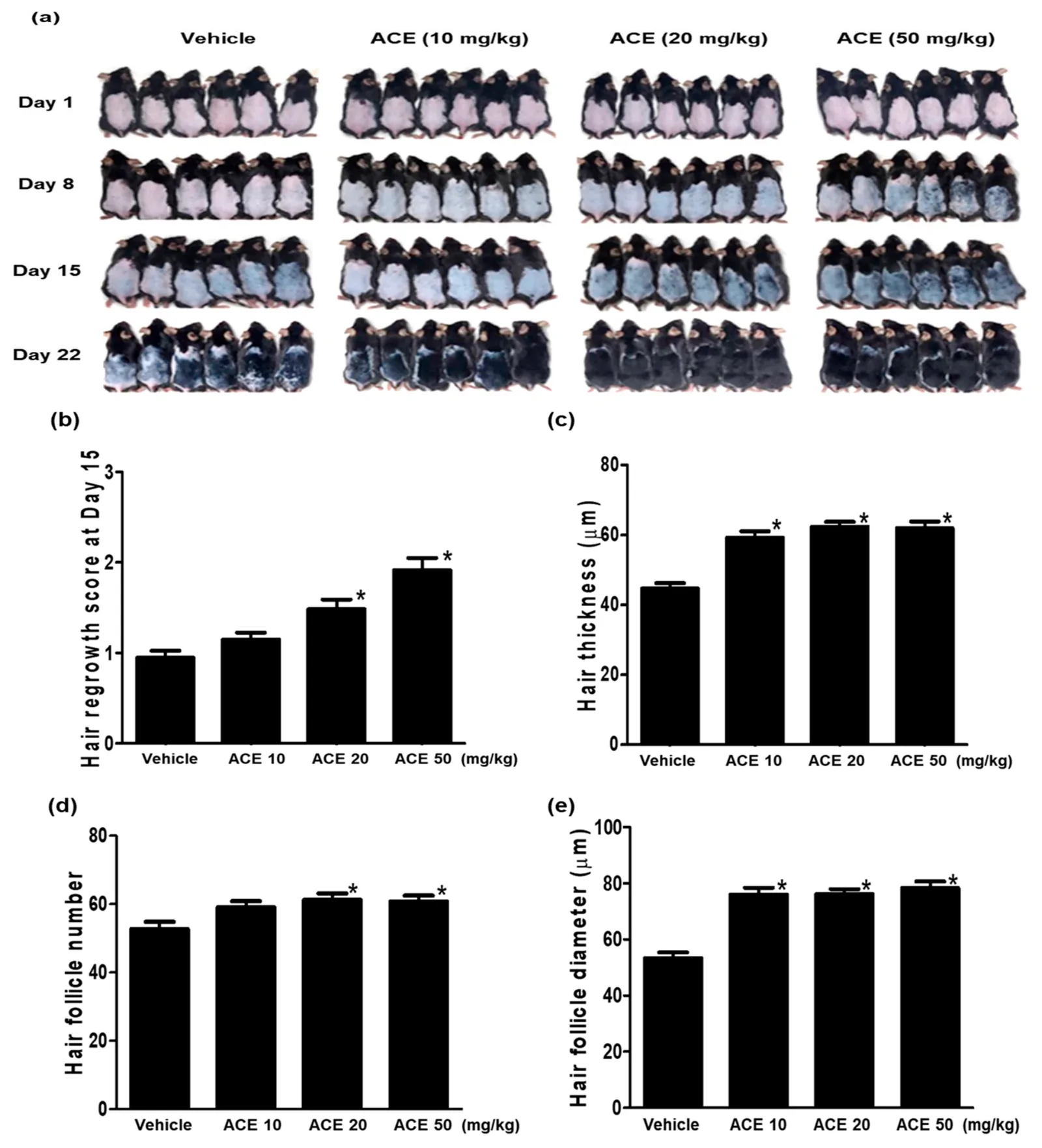
ACE promotes hair regrowth and hair follicle growth in C57BL/6 mice. (**a**) Representative macroscopic images of dorsal skin on days 1, 8, 15, and 22 following daily oral administration of vehicle or ACE (10, 20, or 50 mg/kg; *n* = 6 per group). Skin color progression from pink to black was used as an indicator of anagen-associated pigmentation and hair regrowth. (**b**) Hair regrowth score calculated on day 15 based on the relative area of each skin color score. (**c**) Hair thickness. (**d**) Hair follicle number and (**e**) hair follicle diameter, quantified from H&E-stained dorsal skin sections. Data are presented as mean ± SD. * *p* < 0.05 vs. the vehicle group.

**Figure 2:**
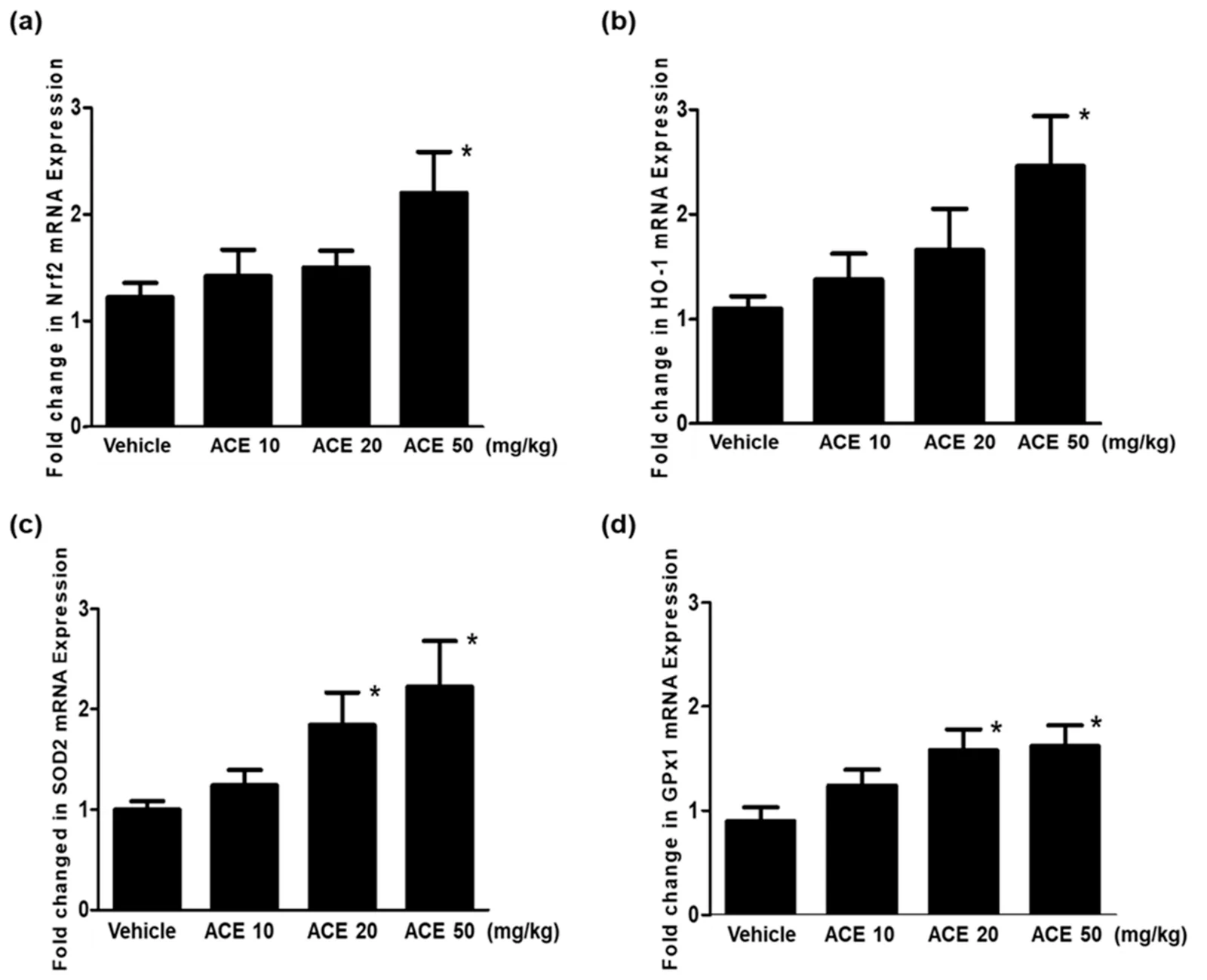
ACE upregulates antioxidant-related gene expression in dorsal skin of C57BL/6 mice. mRNA expression levels of (**a**) Nrf2, (**b**) HO-1, (**c**) SOD2, and (**d**) GPx1 in dorsal skin tissue following 21 days of oral administration of vehicle or ACE (10, 20, or 50 mg/kg; *n* = 6 per group), determined by qPCR and normalized to β-actin. Data are expressed as fold change relative to the vehicle group and presented as mean ± SD. * *p* < 0.05 vs. vehicle group.

**Figure 3:**
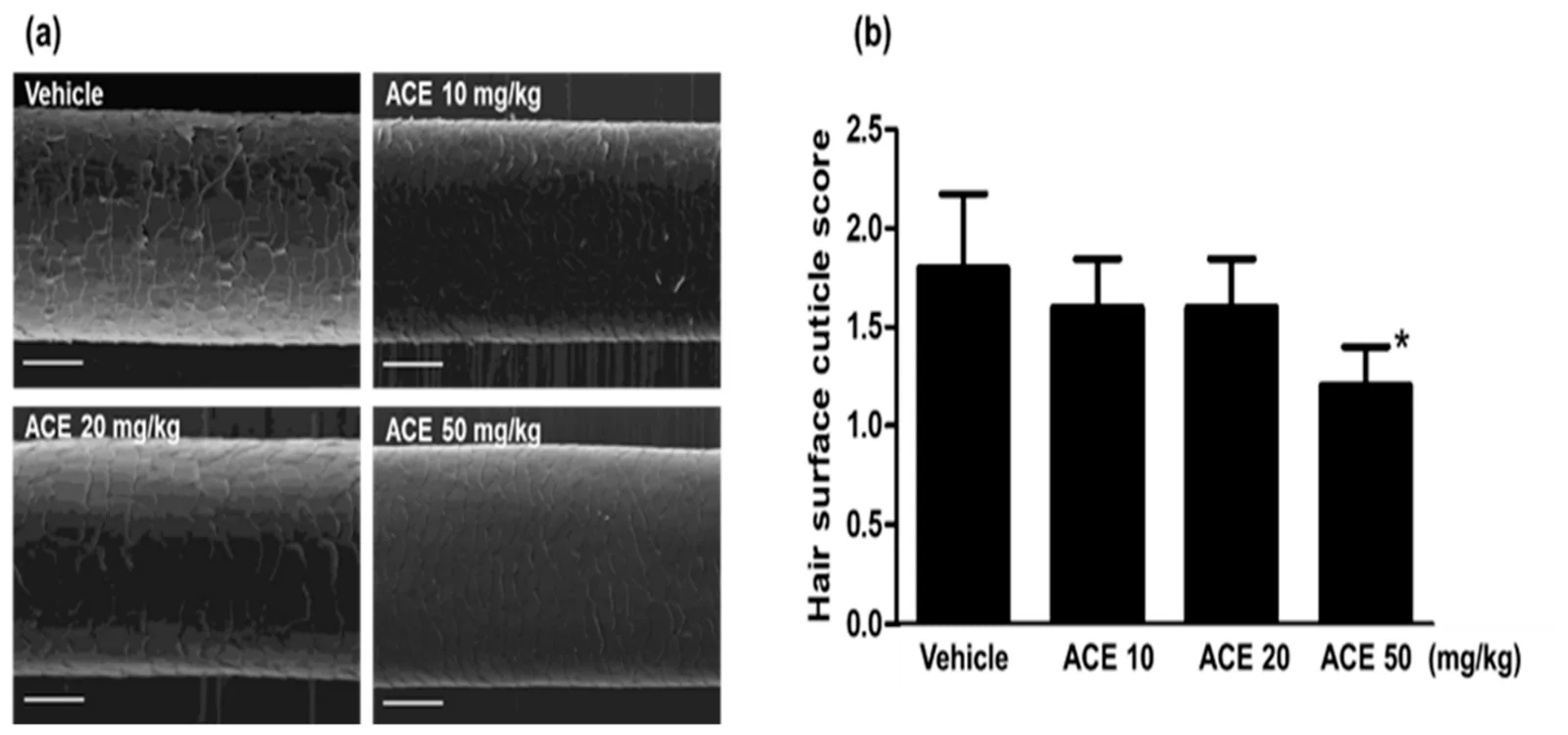
ACE improves hair shaft cuticle integrity in C57BL/6 mice. (**a**) Representative scanning electron microscopy (SEM) images of hair shaft cuticle morphology in the vehicle (**upper left**), ACE 10 mg/kg (**upper right**), ACE 20 mg/kg (**lower left**), and ACE 50 mg/kg (**lower right**) groups. Scale bars = 20 μm. (**b**) Hair surface cuticle score graded on a 5-point scale (Grade 1, intact/undamaged; Grade 5, cortex exposed without cuticle layer) based on SEM images, with higher scores indicating greater cuticle damage. Data are presented as mean ± SD. *n* = 6 per group. * *p* < 0.05 vs. vehicle group.

**Figure 4:**
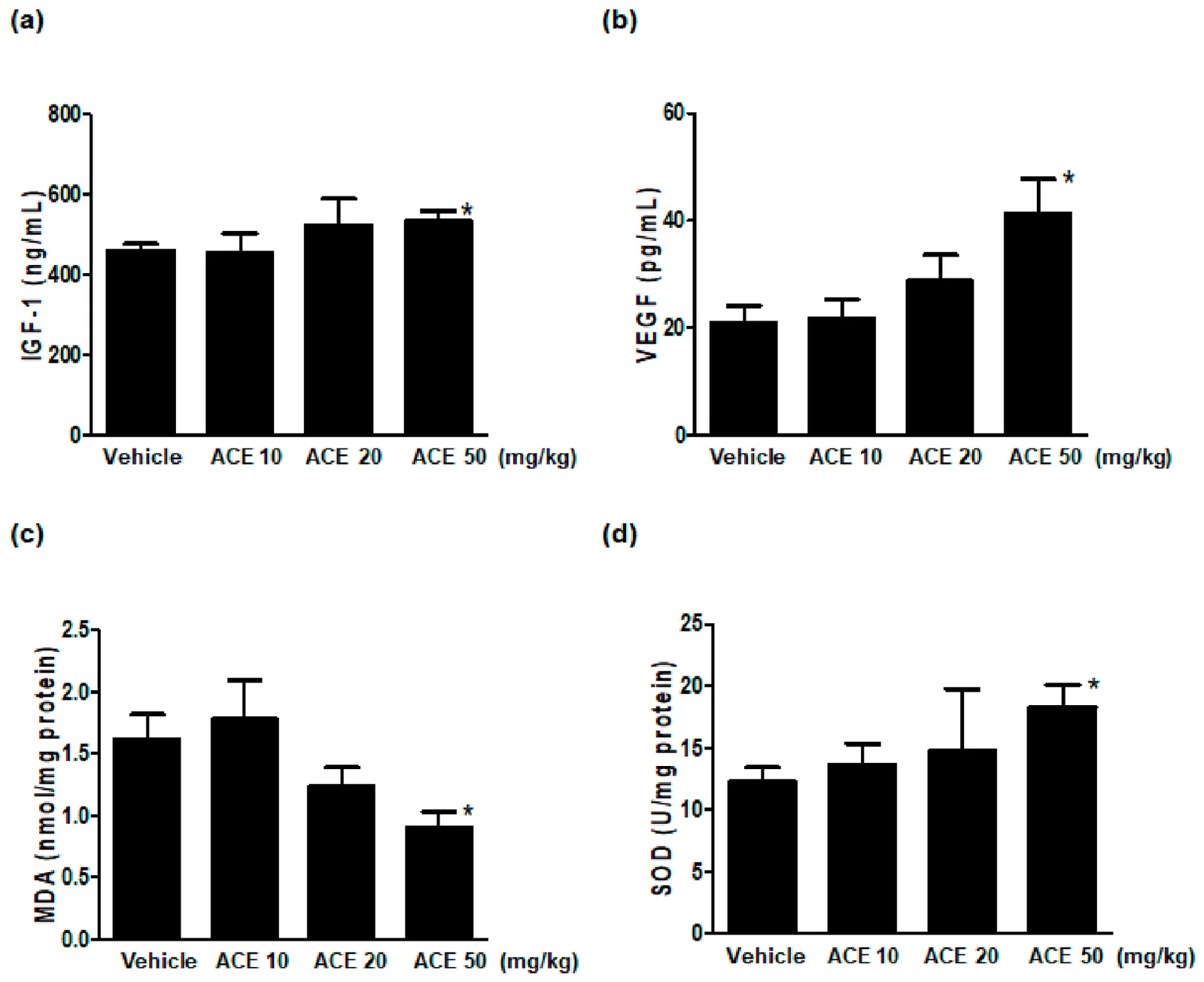
ACE increases circulating growth factor levels and enhances local antioxidant status while reducing lipid peroxidation in C57BL/6 mice. Serum levels of (**a**) IGF-1 and (**b**) VEGF, measured by ELISA; (**c**) malondialdehyde (MDA) levels in dorsal skin tissue, measured by a TBARS assay; and (**d**) superoxide dismutase (SOD) activity in dorsal skin tissue, measured by enzymatic assay, following 21 days of oral administration of vehicle or ACE (10, 20, or 50 mg/kg; *n* = 6 per group). Data are presented as mean ± SD. * *p* < 0.05 vs. vehicle group.

## Data Availability

The original contributions presented in this study are included in the article and Supplementary Material.

## Supplementary Materials

The following supporting information can be downloaded at: https://www.mdpi.com/article/10.3390/cimb48101076/s1.

## Abbreviations

VEGF	Vascular endothelial growth factor
IGF-1	Insulin-like growth factor-1
ACE	*Ageratum conyzoides* extract
Nrf2	Nuclear factor erythroid 2-related factor 2
HO-1	Heme oxygenase-1
SOD2	Superoxide dismutase 2
GPx1	Glutathione peroxidase 1
MDA	Malondialdehyde
SOD	Superoxide dismutase
HF	Hair follicle
HFDPCs	Human follicle dermal papilla cells
SEM	Scanning electron microscopy
HPLC-PDA	High-performance liquid chromatography-photodiode array
IACUC	Institutional Animal Care and Use Committee
H&E	Hematoxylin and eosin
qPCR	Quantitative real-time polymerase chain reaction
RNA	Ribonucleic acid
cDNA	Complementary DNA
mRNA	Messenger RNA
ELISA	Enzyme-linked immunosorbent assay
RIPA	Radioimmunoprecipitation assay
TBARS	Thiobarbituric acid reactive substances
ANOVA	Analysis of variance
MAPK	Mitogen-activated protein kinase
SOD1	Superoxide dismutase 1
GPx	Glutathione peroxidase
GSK-3β	Glycogen synthase kinase 3β
SD	Standard deviation
