# Supplementary material for: Ageratum conyzoides Extract Promotes Hair Shaft Thickness Accompanied by Increased Growth Factor Levels and Enhanced Antioxidant Status in C57BL/6 Mice
Source: Curr Issues Mol Biol. 2026 Oct 18;48(10):1076. doi: 10.3390/cimb48101076 (PMC13703697; doi:10.3390/cimb48101076)
Supplement: Supplementary file 1 [file cimb-48-01076-s001.zip › cimb-4599538_Figure S1.pdf]

Supplementary Materials

***Ageratum conyzoides* Extract Promotes Hair Shaft Thickness Accompanied by Increased Growth Factor Levels and Enhanced Antioxidant Status in C57BL/6 Mice**

Manuscript ID: cimb-4599538

(a) Blank solution

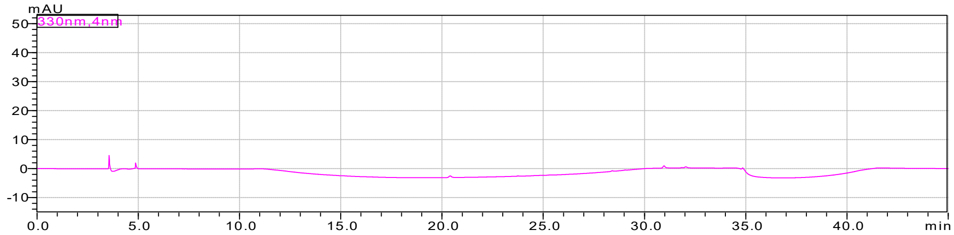

(b) 5′-Methoxynobiletin reference standard

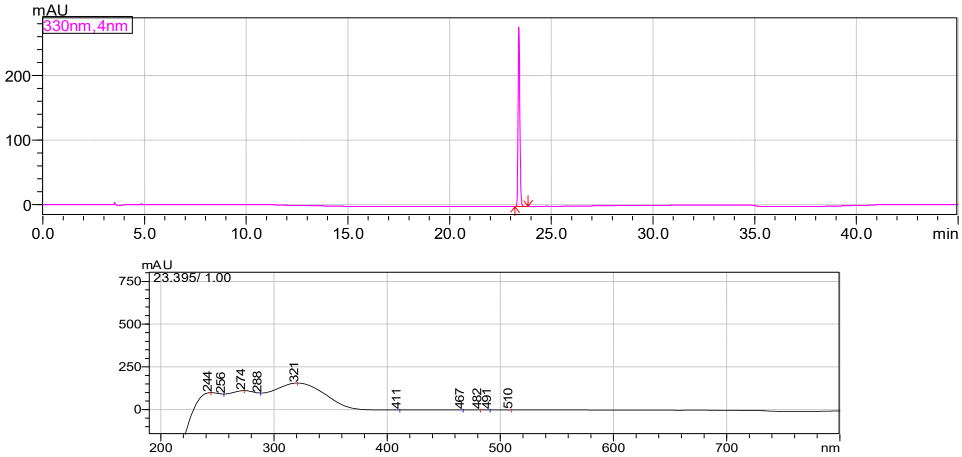

(c) *Ageratum conyzoides* extract (Lot AGE/23002)

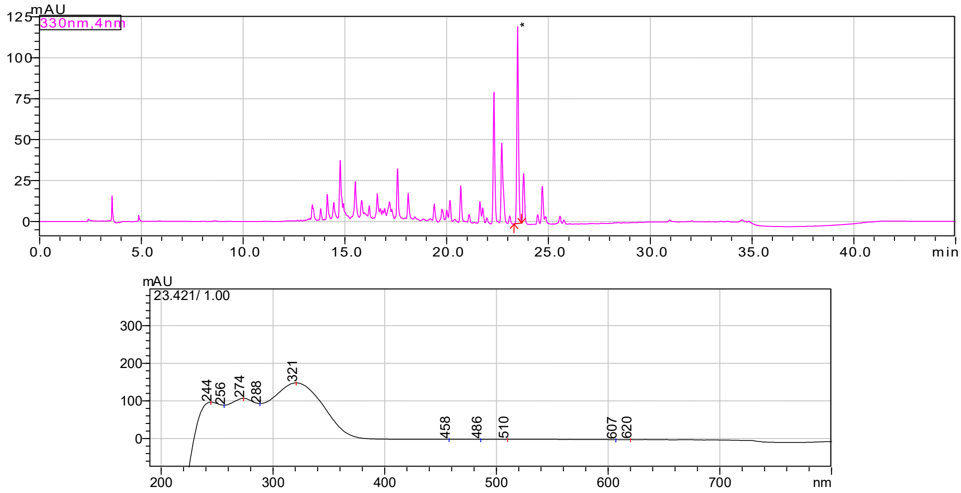

Figure S1. HPLC-PDA chromatograms and PDA spectra for the determination of 5′-methoxynobiletin. (a) Blank solution, (b) 5′-methoxynobiletin reference standard, and (c) standardized *Ageratum conyzoides* extract (Lot No. AGE/23002). Chromatographic detection was performed at 330 nm. The reference standard and extract showed consistent retention times and comparable PDA spectral profiles. The asterisk indicate the 5′-methoxynobiletin peak.
