# Supplementary material for: Ageratum conyzoides Extract Promotes Hair Shaft Thickness Accompanied by Increased Growth Factor Levels and Enhanced Antioxidant Status in C57BL/6 Mice
Source: Curr Issues Mol Biol. 2026 Oct 18;48(10):1076. doi: 10.3390/cimb48101076 (PMC13703697; doi:10.3390/cimb48101076)
Supplement: Supplementary file 1 [file cimb-48-01076-s001.zip › cimb-4599538_Table S1.pdf]

Supplementary Materials

***Ageratum conyzoides* Extract Promotes Hair Shaft Thickness Accompanied by Increased Growth Factor Levels and Enhanced Antioxidant Status in C57BL/6 Mice**

Manuscript ID: cimb-4599538

Table S1. HPLC-PDA conditions, gradient program, and summary of method validation for the determination of 5′-methoxynobiletin in standardized *Ageratum conyzoides* extract.

HPLC-PDA Conditions

Parameter	Condition
Column	Sunfire 5 um C_{18} (250 x 4.6 mm)
Column temperature	35 °C
Mobile phase A	0.1% formic acid in distilled water
Mobile phase B	Acetonitrile
Injection volume	10 µL
Flow rate	0.8 mL/min
Detection wavelength	330 nm

Gradient Program

Time	A (%)	B (%)
Initial	95	5
5	95	5
25	5	95
30	5	95
35	95	5
45	95	5

Method Validation Results

<table>
<tr><th colspan="2">Validation parameter</th><th>Satisfied criteria</th><th>Result</th></tr>
<tr><td colspan="2">Selectivity</td><td>Blank, reference standard, and extract chromatograms and PDA spectra were compared</td><td>No interfering peak;
Consistent retention time and spectral profile</td></tr>
<tr><td colspan="2">Linearity</td><td>Six-level calibration evaluated in triplicate (ranged from 0 to 0.1 mg/mL)
Coefficient of determination (R^2) ≥ 0.990</td><td>Acceptable</td></tr>
<tr><td colspan="2">Accuracy</td><td>Recovery assessment of 3 spiked samples
Mean Accuracy 95–105% (AOAC; 0.1%, 1mg/g)</td><td>Acceptable</td></tr>
<tr><td rowspan="2">Precision</td><td>Repeatability</td><td>Repeat measurements of 5 samples
RSD ≤ 3.7% (AOAC)</td><td>Acceptable</td></tr>
<tr><td>Inter-day precision</td><td>Repeated analyses of 5 samples on different analytical days
RSD ≤ 6.0% (AOAC)</td><td>Acceptable</td></tr>
</table>
